# Development of an antimicrobial peptide-loaded mineralized collagen bone scaffold for infective bone defect repair

**DOI:** 10.1093/rb/rbaa015

**Published:** 2020-04-24

**Authors:** Yuzhu He, Yahui Jin, Xiaoxia Ying, Qiong Wu, Shenglian Yao, Yuanyuan Li, Huiying Liu, Guowu Ma, Xiumei Wang

**Affiliations:** r1 State Key Laboratory of New Ceramics and Fine Processing, School of Materials Science and Engineering, Tsinghua University, Zhongguancun Street, Haidian District, Beijing 100084, China; r2 Department of Oral Prosthodontics, School of Stomatology, Dalian Medical University, Lvshun South Road, Lushunkou District, Dalian 116044, China; r3 Department of Stomatology, Zhejiang Provincial Hospital of Chinese Medicine, The 9th Street, Economic and Technological Development Zone, Hangzhou 310018, China; r4 School of Life Sciences, Tsinghua University, Zhongguancun Street, Haidian District, Beijing 100084, China

**Keywords:** antibacterial peptides, PLGA microspheres, mineralized collagen scaffold, osteogenic differentiation

## Abstract

The repair of infective bone defects is a great challenge in clinical work. It is of vital importance to develop a kind of bone scaffold with good osteogenic properties and long-term antibacterial activity for local anti-infection and bone regeneration. A porous mineralized collagen (MC) scaffold containing poly(d,l-lactide-co-glycolic acid) (PLGA) microspheres loaded with two antibacterial synthetic peptides, Pac-525 or KSL-W was developed and characterized via scanning electron microscopy (SEM), porosity measurement, swelling and mechanical tests. The results showed that the MC scaffold embedded with smooth and compact PLGA microspheres had a positive effect on cell growth and also had antibacterial properties. Through toxicity analysis, cell morphology and proliferation analysis and alkaline phosphatase evaluation, the antibacterial scaffolds showed excellent biocompatibility and osteogenic activity. The antibacterial property evaluated with *Staphylococcus aureus* and *Escherichia coli* suggested that the sustained release of Pac-525 or KSL-W from the scaffolds could inhibit the bacterial growth aforementioned in the long term. Our results suggest that the antimicrobial peptides-loaded MC bone scaffold has good antibacterial and osteogenic activities, thus providing a great promise for the treatment of infective bone defects.

## Introduction

Bone defects induced by infection are a common clinical outcome, the most common of which is osteomyelitis, an inflammatory bone disease. This progressively destructive disease associated with bone loss that could develop following various clinical cases including intravenous drug use, surgical implants, immunodeficiency diabetes and even aging, is dominantly caused by a bacterial infection or infection by other germs, especially *Staphylococcus aureus* [1, 2]. The routine treatment is the combination of local surgery and systemic antibiotic therapy. The defect has a negative impact on aesthetics and functioning after surgical cleaning, hence it is necessary for it to be filled with the bone grafts. With the evolution of biomaterials, there has been a rapid development in the substitute products of autologous or allogenic bone grafts [3]. The artificial bone materials with lower health risks and relatively inexpensive costs have exhibited similar efficacy in promoting osteogenesis, thus providing great potential as replacements for natural bone grafts [4]. At present, many artificial bone graft products, such as ChronOS, Pro-Osteon, Norian SRS, NovaBone and Bio-OSS, are widely used in clinical operations to provide an excellent therapeutic effect for bone defects, but none of them has antibiotic activities. Therefore, to treat the infection in the focal zone in osteomyelitis, the common approach is to administer sufficiently high doses of intravenous and oral antibiotics, which may however result in toxicity [1]. Furthermore, the misuse of a large number of broad-spectrum antibiotics has caused a severe public health problem-bacterial resistance [5, 6]. Therefore, it is important to develop a kind of scaffold with the capability to maintain long-term antibacterial activity locally to kill bacteria and heal the infective bone defects.

Antimicrobial peptides (AMPs) produced from tissues and cells of various invertebrates, plant and animal species, are expected to replace traditional antibiotics because of their non-specialized electrostatic bonding with bacteria. To date, over 2700 peptides with antibacterial properties have been reported, most of which affect the liquid bilayer formation of transmembrane micropores [7, 8]. Based on the action of AMPs on changing the microbe surface charge, membrane proteins, transporters and proteolytic enzymes in the bacteria, AMPs are widely used as antibacterial agents [9–12]. KSL-W (Lys–Lys–Val–Phe–Trp–Val–Lys–Phe–Lys–NH2) and Pac-525 (Ac–Lys–Trp–Arg–Arg–Trp–Val–Arg–Trp–Ile–NH2) are two synthetic antibacterial peptides that have been confirmed to be effective against both Gram-negative and -positive bacteria [13–15]. *Staphylococcus aureus* as the pathogenic bacteria of osteomyelitis and *Escherichia coli* as the representative of common Gram-negative bacteria are used to detect the antimicrobial properties of KSL-W and Pac-525. In previous research, Pac-525 or KSL-W has been loaded in multistage sustained release microspheres or membranes to attain long term and effective antibacterial activity [16, 17]. Because their antibacterial mechanism differs from traditional antibiotics, it is hoped that AMPs will be used as the local antibacterial agents in biomaterials to remedy or replace systemic antibiotics in the future.

However, because of the complexity of the internal environment, AMPs become unstable and easily degraded by microorganisms and proteases in the course of administration, losing their antibacterial activity. Therefore, maintaining the activity and protecting the long-term effect is an urgent problem that needs to be solved for AMPs as an antibacterial component in scaffolds. AMPs could be encapsulated in a degradable sustained release carrier with the advantages of lower mass density, greater surface area and drug release kinetics [18]. Poly(d,l-lactide-co-glycolic acid) (PLGA) has been extensively used as a bioscaffold, biomembrane and drug release carrier in the field of biomaterials because of its high biocompatibility and biodegradability [19, 20]. PLGA microspheres were developed via emulsion or electrospray to encapsulate and deliver drugs in a controlled manner [21, 22].

In this study, we mainly focus on the development of an antibacterial bone scaffold for infective bone defect regeneration by embedding Pac-525- or KSL-W-loaded PLGA microspheres. The bone scaffold we applied here was fabricated via an *in vitro* biomimetic mineralization process to simulate the composition, hierarchy and self-assembled organization of naturally mineralized collagen (MC) fibrils. This biomimetic MC bone scaffold has been proven to possess excellent osteogenic capability and osteoconductivity to promote bone regeneration both *in vitro* and *in vivo* [23, 24].

Here, the physical characterization, biological viability and the antibacterial capability of the antibacterial MC bone scaffold were detected via SEM, swelling and porosity assay, mechanical analysis, cell proliferation and alkaline phosphatase (ALP) activity assay, release measurement and antibacterial activity evaluation. Based on its excellent osteogenic and antibacterial activities, the AMP-loaded MC-based scaffold is expected to be more effective for healing infective bone defects.

## Materials and methods

### Physical characteristics

#### Preparation of AMP@PLGA microspheres

KSL-W- and Pac-525-loaded PLGA microspheres (KSL@PLGA and Pac@PLGA) of biodegradable PLGA were prepared by the electrospraying technique. A mixture of 20 μl AMP solution (150 mg/ml) in 1 ml trichloromethane (THMS) containing 60 mg of PLGA was ultrasonically stirred. The emulsion was placed in a 1 ml syringe connected to a positive voltage (5.23 kV) that was kept at a distance of 20 cm from the receiving plate connected to a negative voltage (1.05 kV). As the injection pump pushed the syringe at the speed of 1 ml/h, THMS was fully volatilized in the process of electrospraying to obtain dry AMP@PLGA microspheres [25].

#### Preparation of the scaffolds with AMPs-loaded PLGA microspheres

To make MC, collagen was dissolved in 0.5 M acetic acid solution followed by the addition of 0.5 M CaCl_2_ solution and 0.5 M H_3_PO_4_ solution, respectively, while fully stirring. Then, the above mixture was added to 0.5 M NaOH solution to obtain the precipitate of calcium phosphate and collagen, which was then washed with deionized water until the pH reduced to 7 [9]. Finally, the precipitate was freeze-dried to obtain the MC.

**Figure 1:**
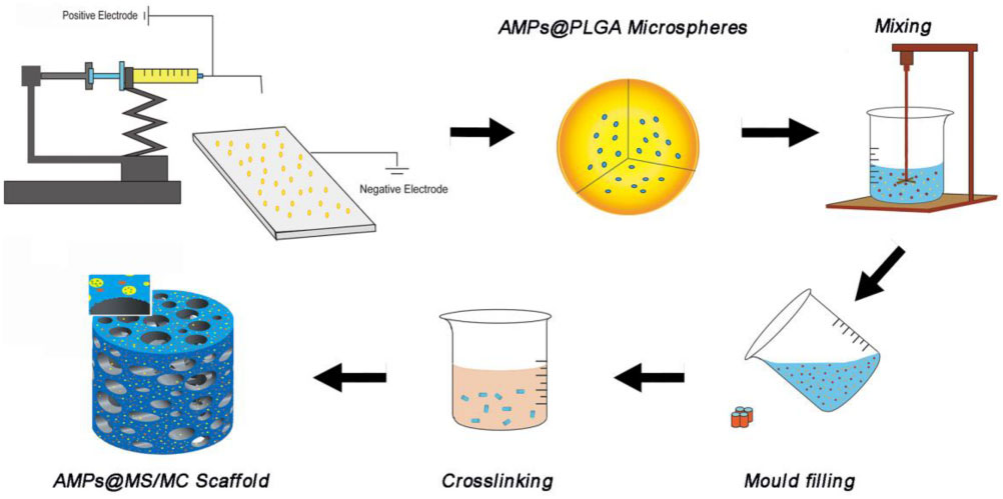
Preparation process of AMP@PLGA/MC.

Collagen (0.5g) was dissolved in 10 ml of 3% acetic acid aqueous solution while stirring overnight; then, the prepared MC (2 g) and AMP@PLGA microspheres (∼100 mg) were slowly added in turn. After full stirring, the mixture was freeze-dried in moulds and then cross-linked by immersing them in 2.5% glutaraldehyde ethanol solution for 24 h. To clear the residual glutaraldehyde, the prepared scaffolds were cleaned with anhydrous ethanol at least six times every 2 h, followed by vacuum drying. Two kinds of AMP@PLGA-loaded MC scaffolds (KSL@PLGA/MC and Pac@PLGA/MC) were obtained for testing (Fig. 1).

#### The morphology of AMP@PLGA microspheres and AMP@PLGA/MC scaffold

SEM (JSM-7001F) was used to detect the size and surface morphology of the fabricated microspheres. Confocal microscopy (Zeiss LSM780) was used to observe the distribution of KSL-W marked with fluorescein isothiocyanate (FITC) in PLGA microspheres. The size of the microspheres and diameter of pores in the scaffold were determined using the Nano Measurer (version 1.2.5.) with the SEM images.

#### AMP embedding rate of PLGA microspheres

PLGA microspheres (10 mg) were dissolved in acetonitrile (0.7 ml) and then added to 1.3 ml phosphate-buffered saline (PBS). After complete dissolution of the microspheres, the drug encapsulation efficiency (EE) of AMP-loaded microspheres was determined by microplate reader (Perkin Elmer EnSpire, USA) at a wavelength of 270 nm with the following formula [26]:

EE （%）=W1/W2×100%,

where *W*1 is the mass of encapsulated AMPs in 10 mg microspheres, and *W*2 is the total mass of AMPs in 10 mg microspheres.

#### Porosity measurement and swelling test

The porosity of AMP@PLGA/MC was calculated using ethanol infiltration because it easily penetrated the pores of composite scaffolds without causing swelling or shrinking as it not a solvent of the polymers. Each weighted sample (*M*1) was sufficiently immersed in a beaker filled with ethanol. Then, the soaked scaffolds were removed from the beaker and their weights (*M*2) were obtained. All operations were finished as quickly as possible. The porosity percentage (*P*) was calculated using the following expression [27]:

P =(（M2-M1)/ρ)/V× 100%,

where *ρ* is the density of ethanol.

The swelling behaviour of the prepared scaffolds was evaluated based on the changes in volume (*V*s) and weight (*W*s). The known volume (*V*1) and weight (*M*1) of the scaffold were immersed in PBS at 37°C for 48 h. The samples were taken out and the volume (*V*2) and weight (*M*2) were measured. While removing scaffolds from the PBS, the excess PBS was absorbed by filter paper. The swelling percentage was calculated using the following expressions [28, 29]:

Vs = (V2-V1)/V1×100%,

Ws = (M2-M1)/M1×100%.

#### Mechanical testing

A mechanical testing machine was used to characterize the mechanical response of the scaffolds in the dry and hydrated states. Axial loading was used on cylindrical samples with height/diameter ratios between 1.5 and 2.0. The samples were placed in the mechanical testing machine with compression speed of 0.5 mm/min. The compressive modulus was obtained as the initial linear slope of the stress–strain relation. The mean of the loaded force acting on the samples was recorded and all specimens were tested three times [30].

### Cell culture

#### Toxicity analysis of AMPs on the MC3T3-E1 cells

The cytotoxicity of Pac-525 and KSL-W was determined by examining their effect on the viability of MC-3T3 cells using the MTT (3-(4,5-dimethylthiazol-2-yl)-2,5-diphenyltetrazolium bromide) assay. The MC-3T3 cells were incubated in 96-well plates with DMEM containing 10% foetal bovine serum (FBS), 1% penicillin and streptomycin at 37°C in a humid atmosphere of 5% CO_2_ for 24 h. For the experiments, MC-3T3 cells were treated by 0, 10, 50, 100, 500 and 1000 µg/ml Pac-525 and KSL-W, respectively, for 24 h. After 4 h of incubation with MTT followed by DMSO for an additional 10 min, the absorbance of each well was measured using the Microplate Reader. To further confirm the influence of these two peptides on MC-3T3 cells, fluorescence staining was used to check the cell morphology. The MC3T3-E1 cells were fixed by 4% paraformaldehyde in PBS for 20 min at room temperature followed by 0.1% Triton X-100 in PBS for 15 min. Then, they were rinsed in PBS three times, stained with TRITC-conjugated-phalloidin for 1 h at room temperature away from light, followed by 4′,6-diamidino-2-phenylindole (DAPI) for 10 min. A laser confocal scanning microscope was used to acquire the images.

#### Cell morphology and proliferation on AMP@PLGA/MC scaffold

The KSL@PLGA/MC, Pac@PLGA/MC and PLGA/MC samples were soaked in α-MEM medium containing 10% FBS and 1% antibiotics (penicillin and streptomycin) ahead of time. Then, MC3T3-E1 cells were seeded on the samples and blank wells for 24 h incubation. The cells were cultured for 24 h in a humidified incubator with 5% CO_2_ at 37°C. Afterwards, the scaffolds with cells were fixed by 2.5% glutaraldehyde solution (v/v) and dehydrated by a certain concentration gradient of ethanol (50%, 75%, 90% and 100%). The refreeze-dried cell scaffolds were used to observe the morphology of MC-3T3 with SEM.

The MC-3T3 cells were cultured on KSL@PLGA/MC, Pac@PLGA/MC and MC scaffolds for 1, 3 and 5 days using the aforementioned method. Then, the cell scaffolds were fixed by the Cell Proliferation Assay Kit (CyQUANT), and DNA content was measured to calculate the number of cells.

#### ALP production activity evaluation

Osteogenic differentiation reflected by ALP activity was induced by osteogenic induction medium with 10 mM β-glycerophosphate, 50 μg/ml ascorbic acid and 10 nM dexamethasone, which was changed every 2 days. The MC-3T3 cells were seeded on KSL@PLGA/MC, Pac@PLGA/MC, PLGA/MC and blank wells; the blank wells were used as the control group. After 14 days of incubation, the MC-3T3 cells grown on scaffolds were harvested and their ALP activity was evaluated using a quantitative protein kit (BIOMIGA) and ALP Elisa kit (NanJing JianCheng Biotech Company). ALP activity levels were normalized to the total protein content of cells at the end of the experiment [31]. All tests were repeated three times.

### Antibacterial efficacy text

#### The antibacterial properties of KSL-W and Pac-525

The mixtures containing 100 μl 10^5^ CFU/ml *S.aureus*/*E.coli* and 100 μl Pac-525/KSL-W solution (5, 10 and 15 μg/ml) were applied to the agar plates. After 18–20 h incubation, the number of bacterial colonies on the plates was observed.

#### Western blot analysis

The effect of Pac-525 and KSL-W on LPS was analysed by detecting the TLR4 level by western blot. The MC3T3-E1 cells were harvested by a modified radioimmunoprecipitation assay buffer (50 mM Tris-Cl, 150 mM NaCl, 1% Nonidet P-40 and 0.1% SDS) with a phenylmethylsulfonyl fluoride and protease inhibitor cocktail. The extracted protein was subjected to electrophoresis on 7.5% SDS–PAGE gel and transferred onto polyvinylidene difluoride membranes (Millipore). After blocking in milk-TBST buffer for 1 h, the membranes were probed with primary antibodies TLR4 (1:1000) and α-tubulin (1:1000) at 4°C overnight. Then, the membranes were incubated in the antimouse secondary antibody IgG (sc-2005) for 2 h at room temperature, followed by visualization with an enhanced chemiluminescence kit.

#### In vitro release

The release behaviours of AMP@PLGA and AMP@PLGA/MC samples were determined by placing them in 2 ml PBS (*Vo*) at 37°C with continuous shaking. At predetermined time intervals (*n* = 1, 2, 3, 4, 5, 6, 7, 14, 21, 28 and 35 days), 1 ml of the initial buffer (*Ve*) was removed for analysis followed by the addition of 1 ml fresh PBS. The amount of AMPs in the release medium was detected using the Microplate Reader at a wavelength of 270 nm. Cumulative AMP release profiles were calculated, and the total amount of AMPs encapsulated in microspheres (*m*) was calculated by the drug EE. The cumulative release rate (*En*) was calculated with the following formula [32, 33]:

$$En\;=\left(Ve\times \Sigma_{1}^{n-1}Cn-1+Vo\times Cn\right)/M\times 100\%.$$

#### Antibacterial activity test

The antibacterial efficacy of AMP@PLGA/MC was evaluated by the Oxford cup disc diffusion method. Two kinds of *in vitro*-release solutions from Pac@PLGA/MC and KSL@PLGA/MC on the 30th day were used to fill up Oxford cups on agar plates cultured with *E.coli* (ATCC 8397) and *S.aureus* (ATCC 25923). After 20 h incubation, the circle sizes around the Oxford cups were recorded to evaluate the antibacterial efficacy of the scaffolds.

### Statistical analysis

All the data are expressed as mean ± standard deviation. The statistical analysis was conducted by one-way ANOVA test using SPSS 13.0 software (Shanghai, China). All data were repeated at least three times. A *P-*value of <0.05 was considered statistically significant difference.

## Results

### Physicochemical properties of the AMP@PLGA/MC scaffold

#### SEM morphology and EE

**Figure 2:**
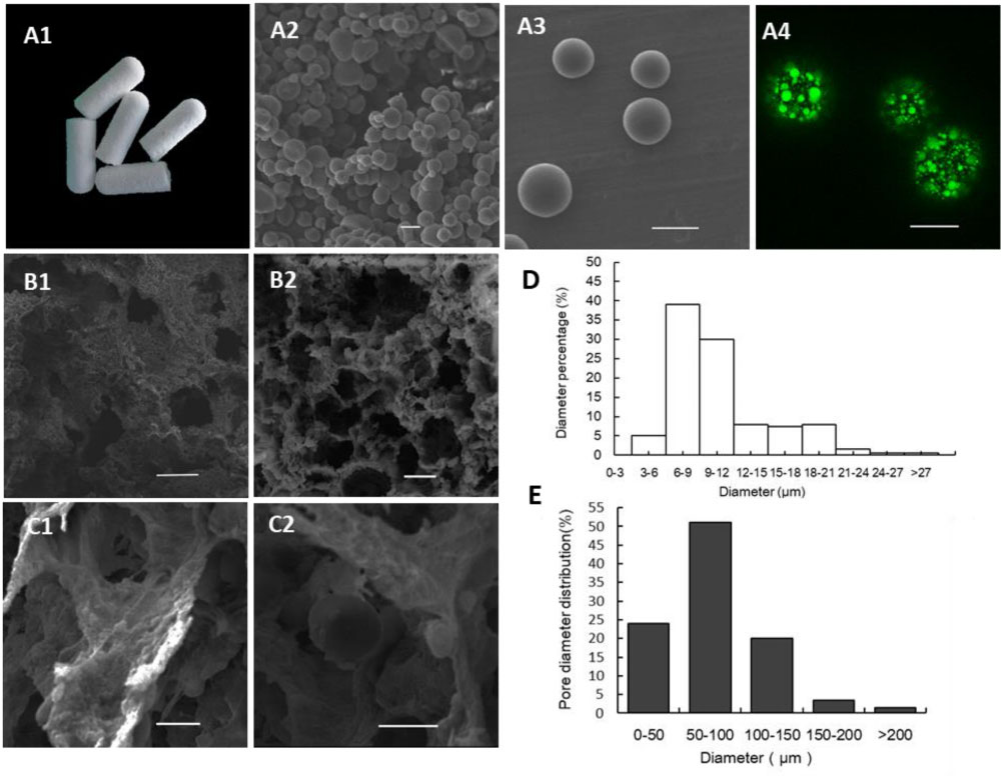
Morphology of AMP@PLGA microspheres and AMP@PLGA/MC scaffolds. (**A1**) AMP@PLGA/MC scaffold. (**A2**, **A3**) Morphology of AMP@PLGA microspheres. (**A4**) The distribution of KSL-W (FITC) in PLGA microspheres. (**B1**, **B2**) The porous structure of MC scaffold. (**C1**, **C2**) MC and AMP@PLGA microspheres on scaffolds. (**D**) The diameter of AMP@PLGA microspheres. (**E**) The pore size of AMP@PLGA/MC scaffolds (**A1–A3**, **C1** and **C2** and **B4** on a scale of 10 µm, **B1** and **B2** on a scale of 100 µm).

The SEM images showed that the microspheres had smooth and compact spherical surfaces and uniform sizes (Fig. 2A2 and A3), the diameter of which ranged from 6 to 12 μm. The location of KSL-W within the PLGA microspheres was visualized with a green fluorescent marker (FITC) that showed an even distribution (Fig. 2A4). The EEs of KSL-W and Pac-525 in PLGA microspheres calculated with the formula were ∼88.93% ± 3.79% and 83.85% ± 6.96%, respectively. The digital photographic image (Fig. 2A1) of AMP@PLGA/MC showed an intact shape with no cracks. The cross section of AMP@PLGA/MC scaffold (Fig. 2B1 and B2) showed a porous structure with MC particles (Fig. 2C1) and PLGA microspheres (Fig. 2C2) homogeneously distributed throughout the scaffold. The pore size of scaffolds was distributed mainly between 50 and 150 μm. The Ca and P content on the surface of the scaffold measured ∼31.94% and 68.06% in weight, which is similar to the actual bones in body (Table 1).

**Table 1:** Elemental analysis

Element	Weight (%)	Atomic (%)
P	31.94	37.78
Ca	68.06	62.22
Total	100.00	100.00

The Ca/P ratio of AMP@PLGA/MC scaffolds.

#### Porosity and swelling tests

The interconnected porous structure was beneficial for cell adhesion and proliferation. A scaffold porosity of 77.57% ± 3.03% was detected with the ethanol infiltration method. The deformation was considered to be an important factor in the application as a bone graft whose excessive volume change would place the surrounding tissues under unnecessary stress. The swelling property of AMP@PLGA/MC scaffolds was investigated by soaking them in PBS followed by the measurement of the change in their weight and volume. Compared with the volume and weight in the dry state, the changes in weight and volume of soaked scaffolds were ∼247.11% ± 27.46% and 7.11% ±1.68% (*V*s), respectively.

#### Mechanical test

**Figure 3:**
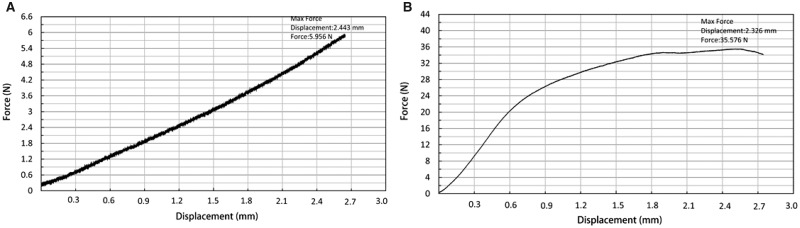
The mechanical property of AMP@PLGA/MC scaffolds. (**A**) The mechanical property of soaked scaffolds. (**B**) The mechanical property of dried scaffolds.

The mechanical property test with axial loading showed that both soaked and dried scaffolds obtained mechanical strength to protect the defect area from being occupied by soft tissue during bone regeneration (Table 2). Compared with the dry state, the compressive strength and elastic modulus of hydrated states were both reduced. According to the stress curve of samples, the deformation of soaked samples in the mechanical test occurred without breaking, which indicates that the soaked scaffolds (Fig. 3A) developed more elasticity than the dried scaffolds that broke under maximum force (Fig. 3B).

**Table 2:** The mechanical strength of AMP@PLGA/MC in hydrated and dry states

Name	Hydrated states	Dry states
Max force (N)	5.32 ± 0.57	26.66 ± 7.73
Compressive strength (MPa)	0.35 ± 0.04	1.85 ± 0.52
Elastic modulus (MPa)	1.01 ± 0.08	18.94 ± 5.51

### *In vitro* biocompatibility

#### Cytotoxicity analysis of AMPs on MC3T3-E1 cells

**Figure 4:**
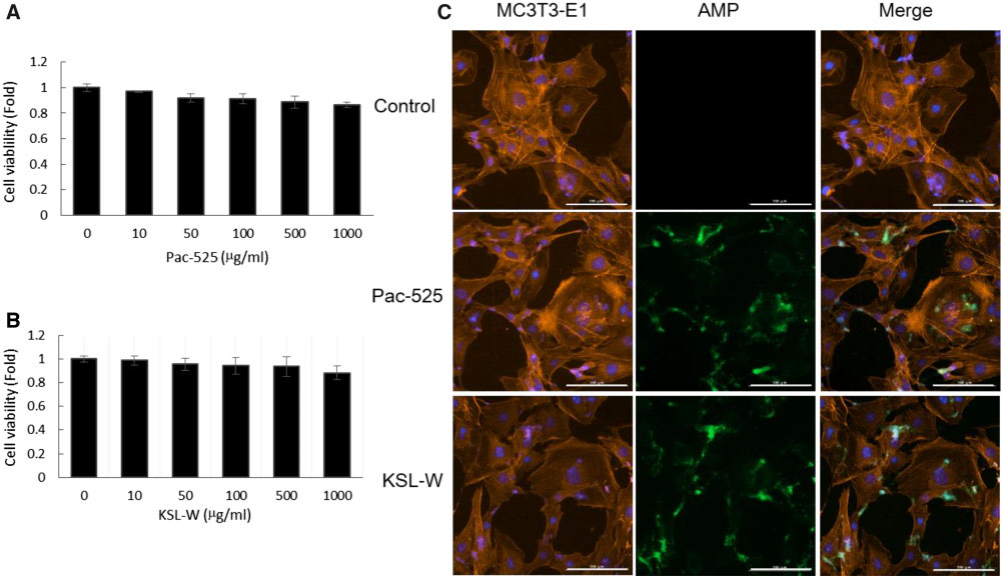
The cytotoxicity of AMPs. The cell viability of MC-3T3 cells treated by (**A**) KSL-W and (**B**) Pac-525 was measured with MTT. (C) MC3T3-E1 cells treated with FITC-AMPs were stained with phalloidin and DAPI (Fig. 4C was on a scale of 100 µm).

The result of the MTT assay showed that at test concentrations from 50  to 500 µg/ml, over 90% of cells survived, which indicated the non-cytotoxicity of the two AMPs (Fig. 4A and B). During fluorescent staining, there was still a small amount of Pac-525 or KSL-W residues on the surface of cells after rinsing with PBS. However, the membrane integrity of MC3T3-E1 cells was not destroyed by the attached Pac-525 and KSL-W, which further proves their non-cytotoxicity (Fig. 4C).

#### Cell morphology and proliferation on AMP@PLGA/MC

**Figure 5:**
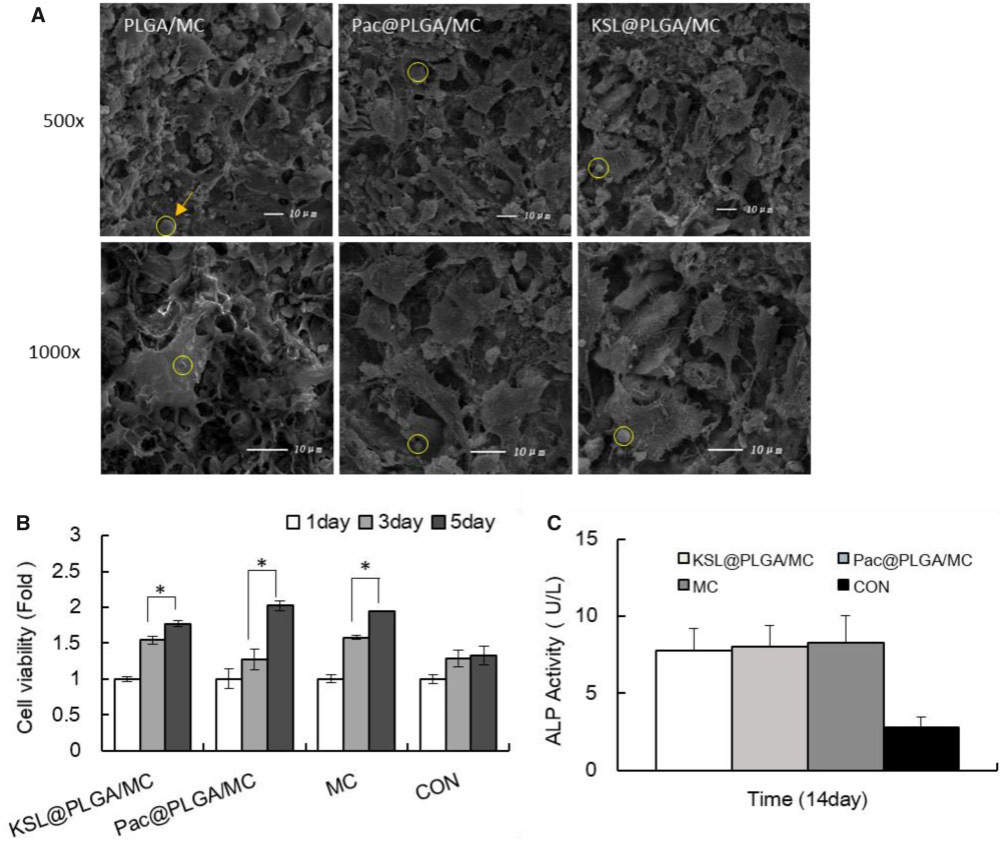
The biocompatibility of AMP@PLGA/MC scaffolds. (**A**) Observation of adhesion and morphology of MC3T3-E1 cells. (**B**) Cell proliferation on scaffolds for 1, 3 and 5 days. (**C**) ALP activity of MC3T3-E1 cultured on scaffolds for 14 days. *P < 0.05. The arrow and circle showed the location of AMP@PLGA microspheres in scaffolds.

The results displayed the morphology of MC3T3-E1 cells, which were cultured on PLGA/MC, KSL@PLGA/MC and Pac@PLGA/MC scaffolds for 24 h. Comparison of Pac@PLGA/MC and KSL@PLGA/MC with MC group showed that MC3T3-E1 cells adhered to the rough surface of the porous scaffolds with the cells fully stretched, and the interlacing process was not affected by Pac-525 and KSL-W (Fig. 5A). The increased cell number of scaffold groups was much more significant than that in the control group, especially in the last 2 days, which indicated the benefit of scaffolds for cell proliferation owing to their porous 3D structure (Fig. 5B).

#### ALP activity evaluation

ALP as the specific sign in the early differentiation of osteoblasts is an important index of osteogenic differentiation ability of cells. The ALP expression of MC3T3-E1 was evaluated on AMP@PLGA/MC scaffolds by using ALP Elisa kit after 14 days of culture, as shown in Fig. 5D. Compared with the control group, the considerable increase in ALP activity in the scaffold group suggests that the osteogenic differentiation promotion by the MC in the scaffold. Furthermore, there was no significant difference between the AMP@PLGA/MC group and MC groups, which indicates that this advantage was not diminished by the addition of AMPs.

### The antibacterial test

#### The antibacterial activity of KSL-W and Pac-525

**Figure 6:**
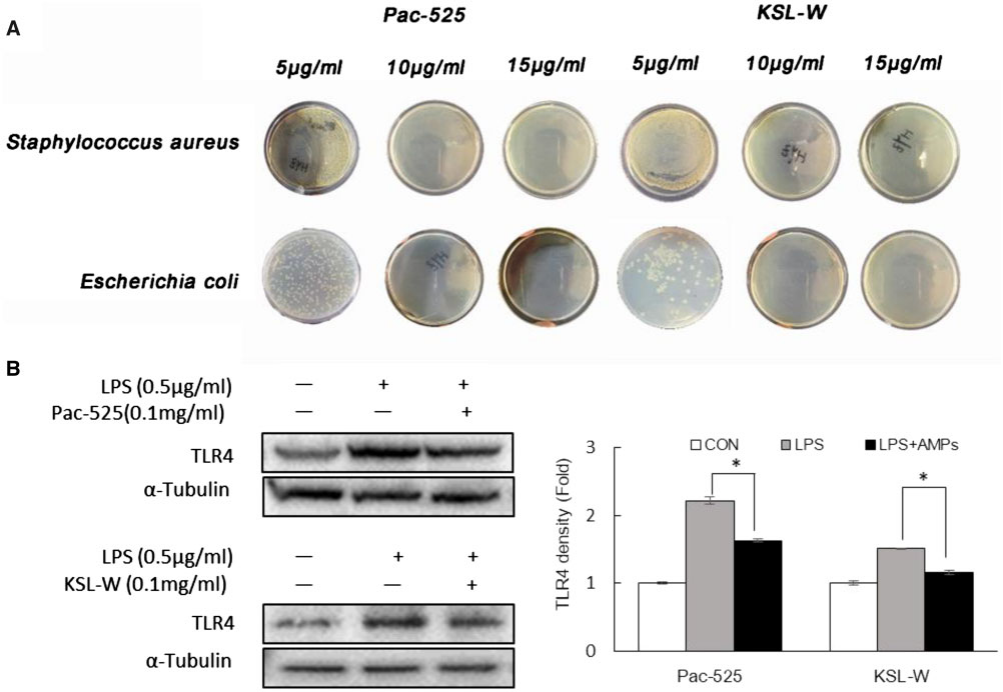
The antibacterial activity of AMPs. (**A**) The inhibition of *Staphylococcus aureus* and *Escherichia coli* by 5, 10 and 15 μg/ml KSL-W and Pac-525. (**B**) Detection of Pac-525 and KSL-W on TLR4 expression in LPS-induced MC-3T3 cells. *P< 0.05.

The antibacterial properties of Pac-525 and KLS-W on *S.aureus* and *E.coli* were treated with certain doses of the KSL-W and Pac-525 peptides in Fig. 6A. With a dose of 5 μg/ml KSL-W and Pac-525, both *S.aureus* and *E.coli* were not completely inhibited. When the dose was increased to over 10 μg/ml KSL-W and Pac-525, the inhibition on bacteria was observed.

As mentioned previously, the AMPs target is the liquid-bacterial membrane bilayer, which mostly consists of LPS in Gram-negative bacteria. TLR4 as an LPS receptor plays an important role in the mechanism of various immune pathways; the TLR4 level reflects the activity of LPS. The result of western blotting showed that the TLR4 level of MC3T3-E1 cells co-treated with LPS and Pac-525 or KSL-W decreased compared with that of the cells that were merely exposed to LPS (Fig. 6B), indicating the interaction between LPS and AMPs.

#### The sustained release of AMPs

**Figure 7:**
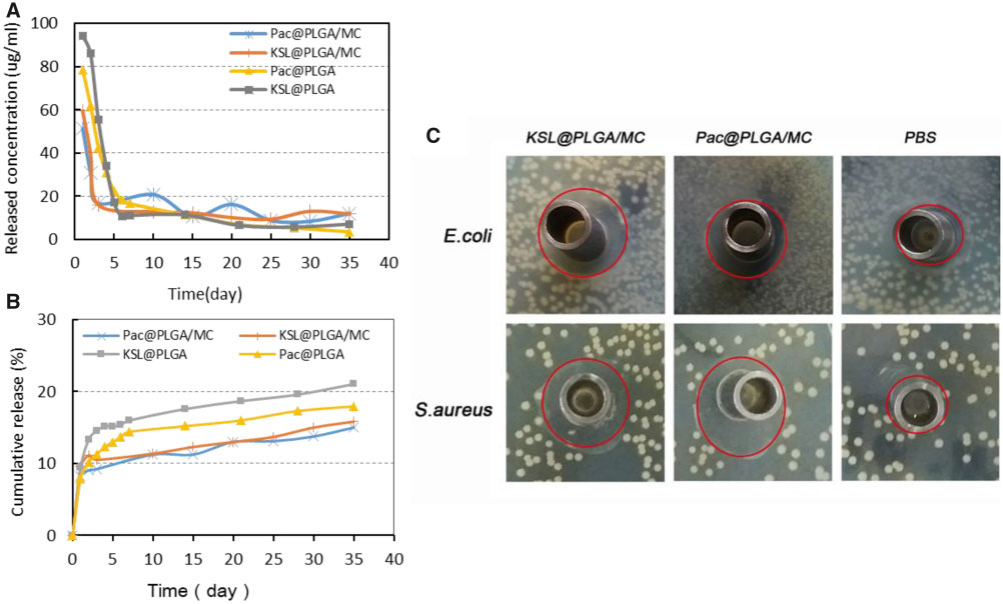
The antibacterial property of AMP@PLGA/MC scaffolds. (**A**, **B**) The *in vitro* release of Pac-525 and KSL-W in KSL@PLGA, Pac@PLGA, KSL@PLGA/MC and Pac@PLGA/MC. (**C**) The clear zone diameters of release solution from Pac@PLGA/MC and KSL@PLGA/MC scaffolds.

The released suspensions of KSL-W or Pac-525 from KSL@PLGA/MC, Pac@PLGA/MC, KSL@PLGA and Pac@PLGA were collected for 35 days. A burst release of AMPs occurred within 2 days in both microspheres and scaffolds, and maintained sustained release in the following days (Fig. 7A). At the end of the incubation period, ∼21% KSL-W from KSL@PLGA, 18% Pac-525 from Pac@PLGA, 15% KSL from KSL@PLGA/MC and 13% Pac-525 from Pac@PLGA/MC were released into the suspension (Fig. 7B).

#### The antibacterial property of released suspensions from AMP@PLGA/MC scaffolds

The suspensions of AMP@PLGA/MC scaffolds released on the 30th day were used to evaluate the antibacterial property by the Oxford cup disc diffusion method (Fig. 7C). The clear zone diameter of AMP@PLGA/MC was significantly larger than that of the PBS group (Table 3), that indicated the antibacterial property of AMP@PLGA/MC. Pac@PLGA/MC had a similar inhibition on both *E.coli* and *S.aureus*, while KSL@PLGA/MC inhibited *S.aureus* more than *E.coli*.

**Table 3:** The effect of KSL-W and Pac-525 released from scaffolds on *Escherichia coli and Staphylococcus aureus*

Name	*E.coli*	*S.aureus*
Bacteriostasis circle size of PBS (cm)	0.97 ± 0.13	0.88 ± 0.02
Bacteriostasis circle size of Pac@MS/MC (cm)	1.32 ± 0.10	1.32 ± 0.09
Bacteriostasis circle size of KSL@MS/MC (cm)	1.33 ± 0.05	1.48 ± 0.12

The clear zone diameters of release solution from Pac@PLGA/MC and KSL@PLGA/MC scaffolds.

## Discussion

Bone defects caused by infection, especially osteomyelitis, should always be treated with surgical therapy and an anti-infection treatment, simultaneously. The additional antibacterial activity of the filling material in surgical therapy could avoid the negative effect of systemic antibiotic therapy and drug resistance. Focusing on this situation, the present work aimed to develop an antibacterial MC-based bone scaffold and investigate the possibility for infective bone defects repair. We demonstrated that the porous scaffold that was loaded with AMP@PLGA microspheres and biomimetic MC provided a sustained antibacterial environment for a guiding structure and component for cell proliferation and osteogenic differentiation.

The main antibacterial agents in the MC-based scaffolds were two kinds of AMPs Pac-525 and KSL-W. Pac-525 and KSL-W have been proven to exhibit antibacterial characteristics on *Porphyromonas gingivalis*, *Fusobacterium nucleatum*, *S.aureus* and *E.coli* [34]. For investigating the property of scaffolds, the release solution for 30 days was used to treat the *S.aureus* and *E.coli*. The diameter of the clear zone suggested that the activities of these two peptides were not decreased by the preparation process (Table 3). Based on the release curve of cumulative concentration in Fig. 7A, after 2 days burst release, the concentration of released peptides was rapidly dropped but remained relatively stable above 10 µg/ml for 2 weeks. With the dose of 10 µg/ml, Pac-525 and KSL-W showed excellent antibacterial activity on *S.aureus* and *E.coli* (Fig. 6A). Furthermore, the antibacterial properties of Pac-525 and KSL-W on bacteria were further proven by detecting the level of TLR4 in MC3T3-E1 cells with western blotting (Fig. 6B). LPS is an endotoxin of Gram-negative bacteria, which has been found to be a target of some AMPs. Therefore, in this study, we hypothesized that the Pac-525 or KSL-W probably could work on LPS to inhibit bacteria. TLR4 is one of the receptors of LPS, expression level of which is related to the amount of LPS in a dose dependent manner [35]. In our study, the decrease in TLR4 in Pac-525/KSL-W and LPS co-treated MC3T3-E1 cells was more significant than that in the LPS only-treated group, indicating the positive effect of Pac-525/KSL-W on targeting LPS. Currently, the mechanism of AMPs to bacteria is not fully established yet. Generally speaking, the actions of AMPs were summarized that they might inherently structure to the target and interact with biomembrane of microorganism via electrostatic interaction and receptor-mediated membrane interaction. Targeting LPS on Gram-negative bacteria may be one antibacterial mechanism of Pac-525 and KSL-W. The rich hydrophobic amino acids included in Pac-525 and KSL-W, such as Val, Ile, Phe and Trp, could aid in their insertion deep into the interior of LPS. Except for hydrophobic amino acid, basic amino acids such as Lys and Arg in Pac-525 and KSL-W played an important role on neutralizing LPS because of their close-packing with the phosphate groups or saccharides of LPS. The fluorescent images in Fig. 4C showed that the Pac-525 and KSL-W attached on the membrane of MC3T3-E1 cells, but the integrity of cells were not damaged. It was further confirmed that the peptide component specifically functioned on bacteria, but not normal cells. While Pac-525 and KSL-W have good broad-spectrum of antibacterial properties on both Gram-positive and -negative bacteria, then other mechanisms such as electrostatic interaction should probably be involved especially on Gram-positive bacteria at the same time. The further studies on the antibacterial mechanism of Pac-525 and KSL-W are still needed.

To retain the activity of Pac-525 and KSL-W in the scaffolds and maintain sustained release, AMP@PLGA microspheres were prepared with PLGA via electrospraying, which was selected based on the purpose, nature of the polymer and hydrophobicity of the drugs. The embedding rate of PLGA microspheres at ∼90% proved that using electrospraying technique successfully reduced the loss of AMPs in the preparation process. The results of *in vitro* release of AMP@PLGA microspheres and AMP@PLGA/MC scaffolds in Fig. 7B showed that during the tested term, ∼30% peptide was released from scaffolds and microspheres, which indicated that the sustained released could be maintained over 35 days. This situation would be beneficial for constructing a long-term local antibacterial environment. Furthermore, the degradation products of PLGA that would cause the local inflammatory response did not affect the adhesion and proliferation of MC3T3-E1 on the AMP@PLGA/MC scaffolds (Fig. 5B). Previously, Yoon *et al.* [36] impregnated de-mineralized bone particles into PLGA scaffolds and found that de-mineralized bone particles effectively reduced the local inflammatory response produced by PLGA degradation. Lickorish *et al.* [37] also reported that the acute inflammatory response to CaP-coated PLGA was significantly less than that of the uncoated PLGA. Although the mechanism was not fully clear, we could speculate that the MC probably helps reduce acid inflammation caused by PLGA degradation.

The MC-based scaffolds showed a porous structure by vacuum freezing, which consisted of an interconnected pore network that lacked regularity (Fig. 1B and C). The porosity of AMP@PLGA/MC scaffolds was ∼77.57% ± 3.03%, similar to that of trabecular bone (70–90% porosity) [31]. On the scale of SEM, the MC3T3-E1 cells adhered and stretched out the parapodium in the pores of the scaffolds. The pore size of the porous scaffold was mainly distributed in the range of 50–150 μm that was expected to contribute to bone regeneration (Fig. 1E), similar to the report by Laurencin *et al*. [38]. The differences in pore size, shape, interconnectivity and porosity of scaffolds would influence cell adhesion, extracellular matrix, cell–cell communications and nutrient transport [39, 40]. It has been reported that the number of cells in the small pore zone increased much more than that in the larger pore zone; however, the larger pores were more beneficial to osteoblast differentiation [41, 42]. ALP is an enzyme whose activity has been observed in the early stages of osteoblast differentiation. The ALP activity assay showed that the ALP activity of MC3T3-E1 cells in MC scaffolds was significantly higher after 14 days of incubation compared with cells in the control group (Fig. 5C). There was no significant difference of ALP activity between the KSL/Pac@PLGA/MC and the MC group. It is suggested that the potential of MC-based scaffolds was not reduced by KSL-W and Pac-525. The results of the osteogenic and antibacterial analysis *in vitro* were helpful to infer the potential of the MC-based scaffold on healing and controlling local bone infection *in vivo*.

In summary, collagen-based scaffolds with osteogenic activity coupled with the sustained release of Pac-525 or KSL-W, are conducive to the realization of a favorable environment that would promote cell differentiation and inhibit bacteria. Even if the antibacterial MC scaffolds achieved the advantage of reducing bacterial resistance by prolonging the peptide action and biological activity, there are still problems to be further studied. In the future, it should be investigated whether these antibacterial scaffolds have the same effect on fungi and viruses, and whether the antibacterial MC-based scaffold would be retained *in vivo*.

## Conclusion

In this study, porous collagen-based antibacterial scaffolds containing electrosprayed AMP@PLGA microspheres and self-assembled MC were designed for bone regeneration in infective bone defects. The analysis of physical characteristics, biocompatibility and antibacterial properties proved that the AMP@PLGA/MC scaffolds showed some benefit on the MC-3T3 cells proliferation and osteogenic differentiation, while also achieving antibacterial activity on both Gram-positive and -negative bacteria. It is predictable that the development of a suitable bone substitute will have a significant impact on the clinical needs in the future.

## Funding

This research was sponsored by the National Natural Science Foundation of China (Grant Nos 31771056, 81671827, 51572144, 61571077, 61871068) and the National Key R&D Program of China (No. 2018YFB0704304).

*Conflict of interest statement*. None declared.
